# Abstracts and Gleanings

**Published:** 1877-06-20

**Authors:** 


### Abstracts and Gleanings.

Elixir Glycyrrhizæ.—By Geo. W. Kennedy, Ph. G.—An elixir by the above name has been introduced in our section within the last few weeks, intended as an adjuvant to disguise and cover the extremely bitter taste of the cinchona alkaloids, epsom salt, and other nauseating and bitter medicines. I can say, after a large number of experiments, that this elixir will admirably answer the purpose for which it is recommended and intended. Experiments made with the view to ascertain and determine the quantity of quinia an ounce of the elixir will completely disguise, prove that the bitterness of from 10 to 12 grains is masked, while with from 15 to 20 grains there is but a slight bitterness observed, comparatively speaking. Hitherto the great objection to quinia as a medicine, especially when given in a liquid form, has been its very bitter taste. There are few sick or convalescent patients who can take it in solution; besides, it is frequen ly prescribed for young children, and to prepare it for those in as palatable a condition as possible, is the great object of elixir glycyrrhizæ, which I hope will fill a vacant place in the line of the many elegant pharmaceutical preparations, and one which I am satisfied, from my own observations, will meet the hearty approval of the medical fraternity.

My object here is to bring this subject again before the medical and pharmaceutical professions and recommend its use, and also to furnish a formula for a preparation which is much pleasanter than simple syrup, and to the former decidedly patateable.

The following formula I find to furnish an excellent elixir:

R. Radic glycyrrhizæ opt......oz. ii,
Spir. vini rect. fort..............f.oz. vi
Aquæ ...............................f.oz. vi
Syr. simplic.......................f.oz. iv
Spir. aurantii.....................f.dr. iss
Spir. cinnamomi ...............min. viii

The spirits are made by dissolving 1 fluidounce of the oil in 15 fluidounces of stronger alcohol.

Make a moderately coarse powder of the root, mix the alcohol and water, moisten the powder with the mixture, allow it to stand twelve hours, pack in a conical percolator, and pour on the balance of alcoholic mixture, and sufficient diluted alcohol, until 12 fluidounces of percolate are obtained, then add the syrup, and finally the spirits of orange and cinnamon.—*Journal Pharmacy.*

[The practitioner may carry this with him labeled *aromatic elixir*, and under that name, using it as a vehicle, can give to children and fastidious patients the most nauseous and bitter drugs without difficulty. Eds.]

CANCER.—Prof. Esmarch, of Kiel, at the late Congress of German surgeons, drew attention to the fact that we are not warranted in dismissing every case of cancer as incurable, where an operation cannot be performed. He recalled the history of a lady who, when declared incurable, took arsenic on purpose to destroy her life. She failed in her design, but cured her disease. He adduced, with illustrative drawings, a large number of patients, the subjects of cancer or epithelioma, or of diseases indistinguishable from them, where large doses of iodide of potassium, or of arsenic, had cured the apparently hopeless malady.—*British Medical Journal.*

BANDAGING FOR ULCERS ON THE LEG.—Mr. Wm. Prowse, in *British Medical Journal*, says that the bandage should always be applied from above, downward. It can be applied more easily, and with greater uniformity and precision, by this method. A bandage thus put on, will keep its place for any required period, without becoming materially slackened. Plasters should be applied in the same way. Cut into strips an inch wide, and apply around the limb from above, downward, each strip being made to overlap, by a third of its width, the preceding one.

APOCYNANUM CANABINUM IN DROPSICAL AFFECTIONS.—Dr. Fentress (in *Med. Brief*) says the above agent is an admirable remedy for the removal of dropsical accumulations, in all cases unattended with inflammatory action. He uses the fluid ext., as follows:

R. Fluid ext. apocynum...dr. ij;
Spts. lav. comp. .......oz. ss;
Syr. simplex...........oz. iijss.

M. One teaspoonful every three or four hours, increasing or lessening the dose, as circumstances indicate. In large doses, it is an active hydrogogue cathartic.

ON THE HYPOTHESIS THAT THE HUMAN OVARIES CONTAIN MALE OR FEMALE OVA. Prof. Mayrhofer, of Vienna, disproves, as he thinks, conclusively, the opinion advanced by Schultze in 1854, and recently supported by Ahlfeld, that the sex of the child is determined by the presence of male and female ova in the human ovary, the order of maturation and impregnation of which is a matter of chance. Mayrhofer believes that the sex is determined during conception by a vital interchange of quality between the ovum and semen.

Preservation of the semen by infrequent coition probably favors the formation of the male sex during conception. Observations in sheep breeding appear to prove the truth of this proposition; the ram begetting male lambs in the beginning of the season—but females later.

It is also exceedingly probable that mammals, cows, for instance, which have become impregnated during the early part of œstruation, more frequently bear female—others impregnated near the end of œstruation, male calves.

An interesting and important question for investigation bearing on this point would be to ascertain the proportion of male and female children in orthodox Jewish families. If the supposition be correct, that among the children of orthodox Jewish parents there are more boys than among the children of Christian parents, the reason would probably be found in the law, which declares the woman unclean during thirteen days from the beginning of menstruation.—*American Journal of Obstetrics*, October, 1876.

[We think it most probable, from observations by stock raisers, that the testicle determines the sex; an ovum fecundated by the spermatazoa from the right testicle producing a male, and the left a female. Ed.]

Dioscoria Villosa, or Wild Yam.—Dr. Goss, of Marietta, Ga., says, of this agent: From the use that I have made of it, I think that it acts directly upon the spinal cord, and the reflex nervous system—the umbilical plexus of nerves specially—and in over-doses it will cause hyperæsthesia of the spinal cord, brain, uterus, and abdominal nerves. But in medical doses, it is one of the most positive remedies we have for bilious colic (so called), which name is very inappropriate to many cases of colic; the vomiting of bile, in many instances, being the result of spasmodic retching. But there are cases of this disease that are caused by vitiated biliary matter; and others from biliary calculi obstructing the gall-duct. It is not only a valuable remedy for spasmodic colic, but also for some cases of neuralgia, especially for those cases that have their seat in the umbilical plexus. Here the pain runs from the hips to the ankles. In dyspepsia, where there is a dull pain in the cardiac region of the stomach, constant belching of gases, cramps of the stomach, or acute lancinating pains in it, then dioscoria will be of much service. In those cases it acts much like the subnitrate of bismuth. It has proved valuable in vomiting, pyrosis, and gastralgia of pregnant females; or, in cases of vomiting during the menstrual flow. In those cases of renal colic from the passage of calculi, which is often mistaken for bilious colic, dioscoria will give prompt relief. Combined or alternated with ipecac, it is a valuable remedy in dysentery, relieving the tormina and tenesmus like opium. In severe attacks of angina pectoris, alternated with the cereus bonplandie, it will be found to give relief where morphia fails. It seems to oppose spasmodic contraction of muscular fibre in the abdominal and thoracic region. To get its benefits, however, it must be fresh, or tinctured while fresh; for, like many good remedies, it soon loses its strength.

Coca.—Dr. Tanner says: Whatever may be said from time to time about the effects of coca on the human system, this much is certain, that it causes timid people, who are usually ill at-ease in society, and particularly so before strangers, to appear to good advantage under these circumstances. In other words, it cures bashfulness. It will certainly prove of inestimable value to that class of people who have made themselves bashful and cowardly by an abuse of the sexual organs, as well as to those who are by nature diffident. It also prevents weariness, either mental or physical, which usually follows prolonged or severe exercise. There is no doubt that it possesses these properties in a high degree, as any one can readily satisfy himself by experiment.

Chronic Chills.—Dr. McNider, in *Brief*, says: I send you a prescription—an infallible one in my hands—for chronic chills, and am sure that no one will be disappointed who will give it a trial:

R. Ferri arseniatis.................... 2 grs.
Quiniæ sulph......................32 grs.
Ext. taraxaci................... .. q. s.
M. Ft. mas. et div. in pil. 32.
S. One three times a day, *after meals.*

The patient should be advised in regard to the arsenic; and when its effects are seen the dose should be reduced to two a day, or even to one.

MIXTURE FOR NERVOUS INSOMNIA, (Graves).

R. Tincture of columbo...... }

Tincture of quassia........ } aa dr. viij,

Tincture of gentian........ }

Tincture of cinchona...... }

Muriate of morphia..........$\frac{3}{4}$ to $\frac{1}{4}$ gr.

M. Three tablespoonfuls a day, in a half cup of tea, one hour before the evening meal, to arrest the nausea and appease the nervous irritability and bring about sleep, particularly in persons who have committed alcoholic excesses—in certain cases lukewarm douches are a useful adjuvant. — *Progressive Medicine*.

EFFECTS OF MEDICINE UPON THE FETUS IN UTERO.—Dr. J. L. Cleveland, in a paper read before the Cincinnati Academy of Medicine, states: 1. That certain remedies, potassium iodide, salicylic acid, and chloroform, may pass from the maternal into the fetal circulation.

The acute exanthemata, scarlatina, measles, small-pox, and perhaps vaccination, can be propagated by the mother to the fetus, whether syphilis passed from the mother to the fetus, or *vice versa*, remains yet undecided.

The effect of maternal, mental, and emotional influences upon the vitality and development of the fetus is undetermined.

As to the therapeutic effects of medicines upon the fetus, almost nothing is known. There is only one class of remedies that are administered with the belief or hope that they will have any effect upon the fetus, viz., syphilis specifics, and the efficacy of these are stoutly denied by some.

Chloroform is known certainly to enter the fetal circulation, but it is not known to exercise any pernicious effects. Zweifel claims jaundice may be caused. This, however, is not proved.

It has not been demonstrated that morphia passes into the fetal circulation, but clinical testimony seems to show that it sometimes does. Clinical experience appears to prove that in the hands of most practitioners, and in the vast majority of cases, opiates may be used with safety to the fetus.

It appears, however, on the other hand, from the testimony of some observers, that the hypodermic use of morphia to its full safe physiological effect produces in the fetus dangerous phenomena, cyanosis, impaired respiration, irregular pulse, contracted pupils, a disposition to sleep, and sometimes convulsions. It is of the utmost practical importance to us all that this latter point should be determined.—*Clinic*.

DIPTHERIA.—Dr. Lucas writes:

*Editor Medical Brief:*

DEAR SIR—Having treated a large number of cases of diptheria after the following plan, and not having lost a case, I briefly give the treatment, in your journal, to the profession. When called to see a case, I make the following prescription, varied according to age, etc.:

R. Fluid ex. baptisia................ 2 drs.
Fluid ex. phytolacea............1 dr.
Hyposulphite of soda............4 drs.
Aqua ..................................4 ozs.

Mix. Teaspoonful every two hours; also use as a gargle every hour or two; rub the neck with a mixture of sweet oil and spirits of turpentine, and then apply fat meat to the throat and, *keep it there*; in bad cases, rub the body all over with lard, at least once a day.

PROGRESSIVE PERNICIOUS ANÆMIA TREATED BY TRANSFUSION.—The patient complained of great weakness, frontal headache, dizziness, impairment of vision and hearing, numbness of fingers, loss of appetite, etc. His condition rapidly became most serious in spite of energetic treatment. Bronchitis set in, and the cerebral symptoms pointed to the risk of sudden and fatal syncope. Under these circumstances transfusion was determined on as a last resort, and it was performed on December 20th. The blood was conveyed from the vein of a healthy man directly to that of the patient by means of Aveling's instrument. The operation lasted about thirteen minutes, and between eighteen and twenty ounces were injected. As soon as the patient manifested uneasiness, the transfusion was stopped; he complained then of a sense of oppression about the chest, but in a little while became more comfortable. The operation was followed by a rise of the temperature, which reached its maximum (103° F.) on the next day, and also by an aggravation of the bronchitis. Ten days after the operation the patient was allowed to get up, and three weeks after he began to take out-door exercise. He was subsequently sent to the Convalescent Hospital, where for sometime he continued to improve. Then the old symptoms gradually returned, and he went home and died about two months after the transfusion. Dr. Glynn regrets that he did not return to the Infirmary, and that transfusion was not again practised.—*The Lancet.*

CROTON CHLORAL IN TOOTHACHE.—Dr. Cleburn writes to the editor of the *Medical Record*, that the specific effect of croton chloral hydrate upon the *fifth* pair in neuralgia suggested its use as an internal and local anæsthetic in toothache. I have derived prompt relief from its use in 5-10 grain doses in the toothache of dyspepsia and pregnancy, and in those aggravating cases which occur in rheumatic and neuralgic patients. Failing to relieve by the ordinary remedies a severe attack of toothache, due to dental caries, I found that a mixture of equal parts of chrystallized cabolic acid, chroton chloral hydrate and solid Japanese oil of peppermint, promptly removed the pain and obtruded all sensitiveness during the process of filling.

GLYCERINE A POISON.—MM. Dujardin-Beaumetz and Audige have made some observations which tend to show that glycerine possesses poisonous properties. Two hundred and forty-five grammes, with an equal quantity of water, injected under the skin, killed a dog in forty-five minutes. Death always follows the injection of .8 of the animal's body-weight. In the only exception where it was recovered from, the animal had drunk large quantities of water. As much as 1.25 gives rise to convulsions of a tetanic kind, which continued till death. There is also increased temperature. sometimes the thermometer reaches 109°. After death, congestion of the cerebrospinal meninges is seen.

FUCUS VESICULOSUS, is a sea-plant, known as '*sea-wrack.*' It has been mentioned as a remedy for obesity, and cases have been reported which seem to establish its efficacy in preventing and removing the deposition of fat in the tissues. The fluid extract is given in doses of a half to one drachm three times per day. It is a good remedy, also, in suppression of the menses.

Laparotomy.—Dr. D.T. Gilliam, Ohio, in *Clinic*, advocates surgical interference in cases of intestinal obstruction, having shown that the mortality, as indicated by the cases thus far reported, is only 20 per cent., while without the operation it is 80 to 90 per cent. He remarks:

"We consider the intestinal and abdominal distention suddenly evolved, with their retinue of morbific influences, as the main element of danger in laparotomy, and as the distinguishing difference between it and ovariotomy.

"The above suggests the necessity of keeping the abdominal distension at the minimum; acupuncture, or aspiration may be of service. Now as to the manner of operating. We do not propose to go into the details of the operation. A few suggestions here, with an application of the same rules relative to the abdominal section in ovariotomy, will be all that is required. Before opening the abdominal cavity, we must not forget the influence of temperature on the vital energies, and ample provision must be made for keeping up the body heat. The middle line is the place to cut; it possesses advantages over every other, and yields the best results. As there is usually much trouble in replacing the escaped viscera, it is advisable to make as short an incision as is compatable with ease of operating. For the same reason it is desirable to prevent the escape of the viscera during the operation. As it is often necessary to withdraw an intussusception before reduction can be effected, Mr. Hutchinson advises that the lower end of the invaginated mass be sought for and withdrawn from the cavity, which will obviate the necessity of removing much bowel from the cavity. In dealing with intussusceptions it is always desirable to use kneading and pressure from the distal end, traction on the ensheathing bowel, and, if need be, much force, rather than to abandon the effort. If the section is made for the purpose of exploration, the search should be systematic and complete. First, make a sweeping exploratron for hernial protrusions; then, commencing at the cœcum or sigmoid flexsure, follow the large bowel in its entire length; returning, make a similar exploration of the small bowel. This may be a little tedious, but it is the only safe way, and is often accomplished while a random search is being carried on with negative results. And here, as we deem it, is the last and most important injunction. After thoroughly closing the abdominal wound and superimposing several layers of cotton-wool, *apply an elastic bandage over all to furnish the requisite presure on the abdominal contents, and prevent the baneful effects portrayed above.*

Sick Stomach.—Frequently we find sick people whose stomachs reject all kinds of nourishment until conditions follow that in many instances terminate fatally. In twenty instances in which we have heard the popular sick-bed nourishments prescribed and rejected by an invalid's enfeebled stomach, we have never known the simple saucer of parched corn, pudding, or gruel refused. The corn is roasted brown, precisely as we roast coffee, ground as fine as meal in a coffee-mill, and made either into mush, gruel, or thin cakes baked lightly brown, and given either warm or cold, clear, or with whatever dressing the stomach will receive and retain. Parched corn and meal boiled in skimmed milk, and frequently to children with summer diarrahea, will almost always cure, as it will dysentery in adults.—Jour. Materia Medica.

A NEW TREATMENT IN POST-PARTUM HEMORRHAGE.—Dr. W. Handsel Griffiths, in the *Practitioner* for March, 1877, speaks thus on the important subject of post-partum hemorrhage: Although not an obstetric practitioner, I have recently been consulted in two cases of severe post-partum hemorrhage. In both cases every means had been adopted, but unavailingly. It flashed across my mind in the first case to try the effect of the ether spray, and accordingly I directed a large spray over the abdominal walls, along the spine, and over the genitals; the uterus at once responded, and the cessation of the hemorrhage was almost immediate. In the second case I lost no time in adopting a similar treatment, and with an equally successful result. I have consulted several eminent obstetric practitioners in Dublin, and am informed by them that they are not aware that this treatment has been heretofore proposed. The advantages of the ether spray over the application of cold water, and the other means usually adopted in these cases,must be patent to every practitioner of midwifery.—*Practitioner.*

SWEET SPIRITS OF NITRE A SOLVENT FOR SALICYLIC ACID.—Dr. D. M. Barkley, Caseyville Ky., writes as follows to the *American Practitioner:* As the administration of salicylic acid has become so extensive, and a good solvent is desirable, I wish to make known, through the your journal, that spiritus nitrici dulcis (sweet spirits of nitre) is the best solvent. I have been prescribing it nearly two years, in the treatment of malarial fevers, with uniform success; in many cases without the use of quinia. I employ this formula:

R. Salicylic acid...............dr. j.
Sweet spirits of nitre......dr. jv M.

Sig.—One teaspoonful every two hours, for children; two to four teaspoonfuls for adults. It can be diluted with water if necessary, and can be combined with veratrum, gelseminum, aconite, etc. One ounce of nitre will dissolve sixteen grains of the pure acid, and make a clear solution.

HYDROCELE.—Dr. Newton, in the *Medical Brief*, says: My plan to prevent the return of the fluid is to follow the injection of equal parts of tincture of iodine and water by close pressure with adhesive plaster, re-applied daily for one week after the operation of tapping, close strapping the testicles upon the pubes. This is done in order that the two surfaces of the membrane may adhere by adhesive inflammation. I have permanently cured many cases that had previously been operated upon by tapping alone, without success. Without the strapping the sack remains open, ready very soon to fill again. By my treatment the sack is permanently closed.

SULPHUROUS ACID IN CHRONIC URTICARIA.—J. V. Shoemaker, A. M,, M. D., in the *Medical and Surgical Reporter*, May 26th, relates a case of this trouble in which he tried all the remedies likely to benefit his patient, among which were alkaline and vapor baths. Different remedies were tried without success, until finally the patient was placed upon one-drachm doses of sulphurous acid in syrup and water three times daily. The patient speedily recovered.—*Practitioner.*

